# Entrepreneurship education revisited: perceived entrepreneurial role models increase perceived behavioural control

**DOI:** 10.1504/IJLC.2017.086856

**Published:** 2017-09-23

**Authors:** Katharina Fellnhofer

**Affiliations:** Lappeenranta University of Technology, LUT School of Business and Management, P.O. Box 20, 53851 Lappeenranta, Finland

**Keywords:** entrepreneurship education, success stories, failure stories, role model, PBC, perceived behaviour control, TPB, theory of planned behaviour, social learning theory, entrepreneurial self-efficacy

## Abstract

Relying on Bandura’s (1986) social learning theory, Ajzen’s (1988) theory of planned behaviour (TPB), and Dyer’s (1994) model of entrepreneurial careers, this study aims to highlight the potential of entrepreneurial role models to entrepreneurship education. The results suggest that entrepreneurial courses would greatly benefit from real-life experiences, either positive or negative. The results of regression analysis based on 426 individuals, primarily from Austria, Finland, and Greece, show that role models increase learners’ entrepreneurial perceived behaviour control (PBC) by increasing their self-efficacy. This study can inform the research and business communities and governments about the importance of integrating entrepreneurs into education to stimulate entrepreneurial PBC. This study is the first of its kind using its approach, and its results warrant more in-depth studies of storytelling by entrepreneurial role models in the context of multimedia entrepreneurship education.

## 1 Introduction

Entrepreneurship education is a crucial element of economic policies aimed at creating employment and growth (Matlay and Matlay, 2006), which has resulted in serious debate about its effects (e.g., Gartner and Vesper, 1994; Henry et al., 2005; Weaver et al., 2006; Dickson et al., 2008; Callagher et al., 2015). A meta-analysis comprising 42 independent samples found that entrepreneurship education was beneficial, presenting a relatively high average effect size (Martin et al., 2013). The analysis included seven studies that measured perceived behaviour control (PBC) as an outcome variable, which was assessed, for instance, by the number of businesses founded (Kolvereid, 1996; Souitaris et al., 2007). Generally, most studies of effects are descriptive and lack a rigorous theoretical framework to evaluate entrepreneurship education initiatives. Several studies have explicitly noted this weakness in the context of effectively measuring entrepreneurship education (e.g., Weaver et al., 2006) and in the context of lack of innovative techniques for increasing entrepreneurial behaviour (e.g., Kuratko, 2005; Chen, 2014).

Although 19 studies in the literature have applied the theory of planned behaviour (TPB), originally conceptualised by Ajzen (1988), only a few have used entrepreneurial behaviour as a dependent variable (e.g., Souitaris et al., 2007). In addition, existing research has neglected the effects of entrepreneurial education and entrepreneurial role models as facilitators of entrepreneurial career choices or behaviour (Muofhe and du Toit, 2011). Therefore, to help fill this gap, the present study aimed to investigate the effects of role models on entrepreneurial PBC in both outside and in the context of entrepreneurship education. To ensure the generalisability and validity of the applied mechanisms and techniques, the study built on three theories: TPB (Ajzen, 1988), social learning theory (Bandura, 1986), and Dyer’s (1994) model of entrepreneurial careers (Glaub et al., 2014).

The validity of TPB has been stressed in previous studies, including those by Sheppard et al. (1988), Krueger et al. (2000), Krueger (2009), and Schlaegel and Koenig (2014). According to Liñán et al. (2011), perceptions favouring an action – in this case becoming an entrepreneur – which is defined as PBC, can be influenced in the context of entrepreneurship education. In particular, a central aim of most entrepreneurship programs is to create awareness of entrepreneurial activities (von Graevenitz et al., 2010) or necessary entrepreneurial knowledge and skills (Oosterbeek et al., 2010). In this regard, social learning theory (Bandura, 1986, 1977) provides the fundamentals of how entrepreneurship education can affect entrepreneurial behaviour. Bandura’s (1977) theory emphasises the importance of observing others to discover new knowledge and paths by observational learning. Finally, the present study also builds on Dyer’s (1994) model of entrepreneurial career choices, which stresses the positive contribution of role models. One such contribution, perceived self-efficacy, is a central variable in the framework proposed by the present study.

In the context of self-efficacy also entrepreneurial experience via knowledge plays a role. For instance, Li et al. (2015) stress that prior knowledge significantly and indirectly affects entrepreneurial opportunity recognition via its impact on entrepreneurial alertness. Furthermore, a longitudinal survey on behalf of Roxas (2014) comprising data of 245 students in a Philippine University observes the direct and indirect effects of knowledge on an individual’s entrepreneurial intentions increased via an entrepreneurship education program which highlights the importance of emerging knowledge to ones’ self-confidence and an attitudinal tendency toward entrepreneurship. Additionally, Malebana’s (2017) findings stress significant associations between the knowledge of entrepreneurial support and entrepreneurial intention among others. This result derived from a sample consisting of 355 students from two African universities. Thus, role models are expected to accelerate ones’ entrepreneurial knowledge and experience.

In general, entrepreneurial experience and knowledge are based on entrepreneurial learning which has established to a topic of substantial interest (McKeon et al., 2004). Entrepreneurial learning tends to be practitioners related. It can be multifaceted extending from courses to more structured degree programs. For instance, McKeon et al. (2004) discuss an example how multinational enterprises provide a crucial source of learning for small to medium-sized enterprise entrepreneurs. Thus, following this vital learning tree, existing entrepreneurs could act as role models and thus a source of entrepreneurial learning for future entrepreneurs. However up to now, in particular, research dedicated toward multimedia entrepreneurial learning of nascent entrepreneurs is limited. With the objective of extending our understanding in this discipline, this investigation inspects role models as a source of learning for potential entrepreneurs. Role models show potential to build bridges for innovative entrepreneurial activities such as consultants in technology transfer (Bessant and Rush, 1995) or to implement a mechanism for strengthening enterprises (Ianioglo and Polajeva, 2017) in our changing society (Drucker, 2011).

Entrepreneurial behaviour as an outcome of entrepreneurship education warrants more intensive research attention (Pittaway and Cope, 2007; Bird et al., 2012). The present study assessed the effects of role models in entrepreneurship education by embedding multimedia narratives of entrepreneurs discussing their successes and failures in a web-based questionnaire. The results of regression analysis are based on 426 individuals, primarily from Austria, Finland, and Greece, show that role models increase learners’ entrepreneurial perceived behaviour control (PBC) by increasing their self-efficacy.

The paper is structured as follows. Section 2 discusses the study’s theoretical framework. Section 3 discusses its hypotheses regarding the effects of entrepreneurship education on entrepreneurial PBC. Section 4 discusses the study’s methodology, and Section 5 presents the results of its regression analyses. Section 6 discusses the study’s conclusions and practical implications, including its limitations, and suggests further research.

## 2 Theoretical framework

Entrepreneurship education is “any pedagogical program or process of education for entrepreneurial attitudes and skills” (Fayolle et al., 2006, p.702). Within this framework, there are various types of objectives for various groups targeted by entrepreneurship education (McMullan and Long, 1987; Gorman and Hanlon, 1997; Bridge et al., 1998; Liñán, 2004), which focuses mainly on increasing awareness of entrepreneurship, but which should also prepare potential entrepreneurs for founding new ventures. Entrepreneurship programs that seek to do this disseminate entrepreneurial knowledge and skills to help potential entrepreneurs start businesses (e.g., Liñán, 2004; Boyles, 2012) or motivate their entrepreneurial talents (Glaub et al., 2014) via entrepreneurial coaching (Rasmussen and Sorheim, 2006). Other entrepreneurship education studies have focused on programs that increase confidence related to performing various entrepreneurial tasks (e.g., Chen et al., 2001).

Prior studies have indicated that multimedia communication by entrepreneurial role models that aim to facilitate entrepreneurial behaviour have great potential to present entrepreneurism as an attractive career path (EC, 2013). In addition, prior research has indicated that investigating this type of education requires theoretical fundamentals, including TPB (e.g., Robinson and Sexton, 1994; Kolvereid and Moen, 1997; Marques et al., 2012; Rauch and Hulsink, 2015) and social learning theory (Bandura, 1986) with respect to prior research linked to gender-related issues (e.g., Anna et al., 2000), entrepreneurial identity (e.g., Matlay and Harmeling, 2011) or role models (e.g., Chlosta et al., 2012). These approaches make up a wide-ranging portfolio of diverse education techniques (Kuratko, 2005; Mustar, 2009; Neck and Greene, 2011). Entrepreneurship courses not only facilitate acquiring entrepreneurial skills, but they also aim to inspire, motivate, and positively affect perceptions of entrepreneurship; in other words, to stimulate entrepreneurial PBC.

### 2.1 Theory of planned behaviour

Overall, the concept of TPB has been applied across different disciplines. For instance, TPB was used for predicting the intention to marriage (Shahrabadi et al., 2017), cycle commuting intention (Lois et al., 2015), job seekers’ intention (Tsang et al., 2015), intention of females’ breastfeeding in areas of economic hardship (McMillan et al., 2008) or farmers' decisions to diversify or specialise their businesses (Hansson et al., 2012). To be more precise, Ajzen’s TPB (1988) proposes that (entrepreneurial) behaviour is best predicted by (entrepreneurial) intentions to perform the anticipated (entrepreneurial) behaviour one day (Ajzen, 1988, p.132) and that such intentions are formed by attitudes, subjective norms (SN), and PBC. Attitudes are defined by perceptions that a particular (entrepreneurial) behaviour will lead to an expected result. SNs are reflected by the perceptions of others in the social environment, including family, friends, and role models regarding a particular behaviour. PBC presents perceptions concerning entrepreneurial behaviour as self-controllable. Several studies have applied TPB to evaluate both the likelihood of becoming an entrepreneur (Krueger et al., 2000; Peterman and Kennedy, 2003; Liñán and Chen, 2009; Kautonen et al., 2013) and the effects of entrepreneurship education (Athayde, 2009; Mwasalwiba, 2010; Liñán et al., 2011; Ferreira and Fernandes, 2012; Schlaegel and Koenig, 2014). Therefore, TPB provides a profound, well-validated framework for assessing in detail the effects of entrepreneurial role models on entrepreneurial behaviour. However, TPB alone represents an insufficient condition for improving entrepreneurial culture and engagement. Another important aspect is entrepreneurial socialisation.

### 2.2 Social learning theory

Another theoretical approach highlights the role of entrepreneurial socialisation. Social learning theory (Bandura, 1986, 1977) is conceptually narrower than TPB and thus provides a basis for how entrepreneurship education affects entrepreneurial PBC by influencing the motivation and capability to engage in specific entrepreneurial activities. Bandura’s (1977) theory emphasises observing others’ emotions, attitudes, and behaviours. In short, learning by observing the environment enables discovering new knowledge and paths. In this framework, the multi-dimensional concept of entrepreneurial self-efficacy is a strong tool for understanding the driving force for creativity (Tierney and Farmer, 2002), which has been discussed in the context of entrepreneurship education (e.g., Lerner et al., 1997; Klapper, 2014).

### 2.3 Entrepreneurial career model

Dyer’s (1994) model of entrepreneurial careers explains the components of education central to preparing individuals for a successful entrepreneurial career, including career selection, socialisation, orientation, and development (Gibb, 1994). Based on this model, entrepreneurial career choices, among others, can be influenced by role models. In the framework of the present study, a central variable was perceived self-efficacy, which affects individuals’ expectations regarding future outcomes that influence career goals. Several qualitative studies have investigated the relationship between education and career choices linked to entrepreneurship (e.g., Albert et al., 1991; Lerner et al., 1997; Dickson et al., 2008; Solomon and Matlay, 2008; Stokes and Wilson, 2010).

In short, TPB, social learning theory and entrepreneurial career model are important underlying theoretical concepts that role models, enhanced with their success and failure stories (communicated by multimedia), can facilitate entrepreneurial self-efficacy via PBC in entrepreneurship education. While (entrepreneurial) perceived behaviour appears to be an adequate predictor according to Ajzen’s TPB (1988), Bandura’s (1977) theory emphasises observing others effect (entrepreneurial) self-efficacy which is also in line with Dyer’s (1994) model of entrepreneurial careers stressing that education – via observing and learning from role models – can boost one’s entrepreneurial knowledge and experience to increase PBC which appears to be crucial when preparing individuals for a successful entrepreneurial career.

## 3 Hypotheses

**Figure 1:**
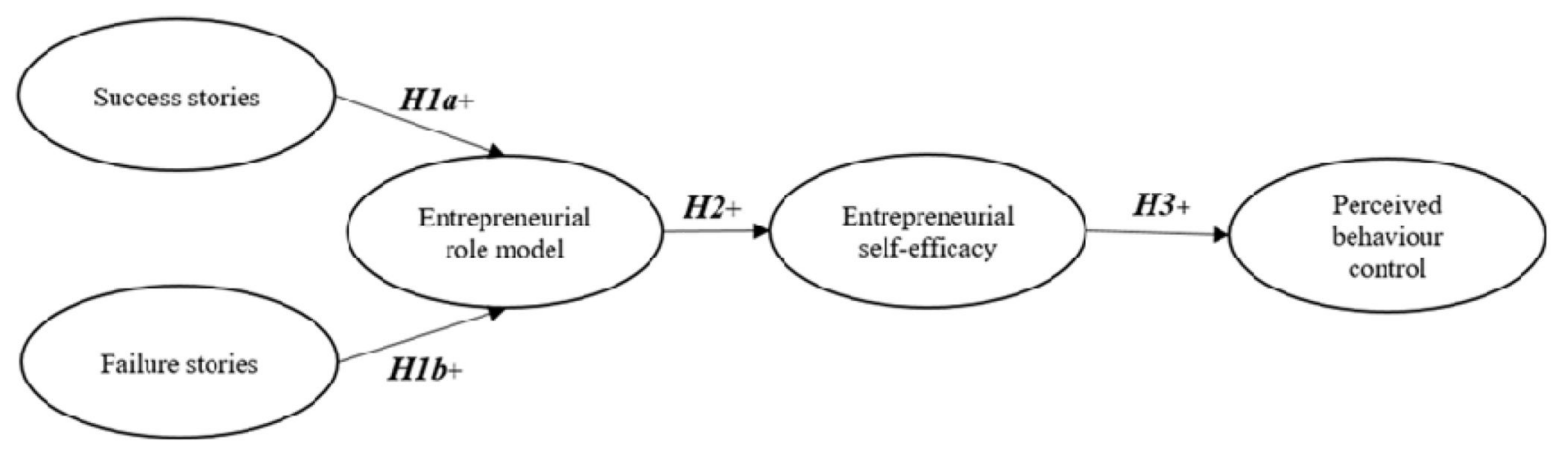
Proposed research model

Entrepreneurship education shows great potential to boost entrepreneurial PBC by persuading students to establish businesses (Fayolle et al., 2006). The present study developed an integrative model that provides a basis for a measuring instrument that builds on Ajzen’s (1988) theory and also integrates elements of other theories, including social learning theory (Bandura, 1986, 1977), and career models, including Dyer’s (1994) model of entrepreneurial careers. Since these models are associated with entrepreneurial career choices and appear to be interrelated, an integrative research model was developed for this study. Figure 1 depicts the integrative research model, which does not explicitly include SN and attitudes, although they reflect crucial elements of the original TPB. To stress the underestimated potential of role models in entrepreneurship education, the study defined ‘SN’ as role models and ‘attitude’ as entrepreneurial self-efficacy that represents antecedents of PBC. In other words, the study assumed that role models, enhanced with their success and failure stories (communicated by multimedia), facilitated entrepreneurial self-efficacy via PBC in entrepreneurship education. The hypotheses underlying this assumption are discussed next.

### 3.1 Success and failure stories

Although the notion of the entrepreneurial role model was first introduced by Gibson (2004) as a cognitive construction to similar identities who are attractive to imitate, so far its effects on and relationship to entrepreneurship education have been not studied. According to Lockwood (2006), role models are persons who present an example of the desired success and thus a template of the behaviours required to be successful. Some studies (e.g., Bosma et al., 2012; Chlosta et al., 2012) have shown that parents who are entrepreneurs act as entrepreneurial role models and increase entrepreneurship behaviour in their children by the socialisation process in the family setting (Bandura, 1977). Entrepreneurial role models can also be outside the family. Kuckertz (2013) and others (Aronson, 2004; Souitaris et al., 2007; Carver et al., 2010) have underlined the educational advantages of observing such individuals. Thus, in line with the recommendations of Rahman and Day (2014) on informal and formal learning, role models who tell entrepreneurial stories would strengthen the traits and personalities of potential entrepreneurs. Within this narrative context, what the entrepreneurs did in certain situations is of interest (Harmeling and Sarasvathy, 2013) and shapes perceptions of them as role models. In principle, biographic assignments (Verduyn and Jansen, 2005) have already been applied as a pedagogical technique in entrepreneurship education. Overall, sharing professional stories facilitates the learning process in multiple ways. Practical knowledge is constructed through understanding and interpretation, and as a consequence, a bridge is built between theory and practice (Ritchie and Wilson, 2000; Dewhurst and Lamb, 2005). Because storytelling develops reflective and explanatory thinking, this educational method appears to be useful for constructing (entrepreneurial) knowledge, identity, and skills in both the listeners and tellers (Schatz-Oppenheimer and Dvir, 2014). In short, in entrepreneurship education, both the success and failure stories of entrepreneurial role models show great potential for inspiring one’s perceptions of role models. Thus, we predicted the following.

H1a: Entrepreneurial success stories influence one’s perceptions of role models.

H1b: Entrepreneurial failure stories influence one’s perceptions of role models.

### 3.2 Entrepreneurial role models

According to some studies (Aldrich et al., 1998; Hout and Rosen, 1999), by serving as role models, entrepreneurial parents, family members, or friends influence career choices and self-efficacy. General self-efficacy relates to “one’s estimate of one’s overall ability to perform successfully in a wide variety of achievement situations, or to how *confident* one is that she or he can perform effectively across different tasks and situations” (Chen et al., 2001, p.63). In this context, several studies, in particular, have found that self-efficacy increases entrepreneurial intentions (e.g., Zhao et al., 2005; Wilson et al., 2007; Pihie and Bagheri, 2013) and motivations to found a business (e.g., Chen et al., 1998; Rauch and Frese, 2007; Lin and Si, 2014). Both researchers and educators have demonstrated that perceived self-efficacy influences behaviour, and entrepreneurial self-efficacy plays a critical role in motivating individuals to become entrepreneurs. Fundamentally, Bandura (1986) concluded in his empirical study on entrepreneurial education and self-efficacy that entrepreneurial education positively affects perceptions of one’s ability to become an entrepreneur. In addition, Fayolle et al. (2006) specified that entrepreneurial intentions are more solid when self-efficacy is increased by the attentions of entrepreneurial role models. In their multiple regression analyses, Quimby and Santis (2006) found that levels of self-efficacy and role-model inspiration differed across various career paths. In line with these studies, the present study expected that exposure to entrepreneurial role models within the framework of entrepreneurship education would increase confidence in the overall ability to follow an entrepreneurial career path. Overall, results of the present study were expected to be in line those of Rahman and Day (2014), who argued that role-model involvement in entrepreneurship education increased motivation to choose an entrepreneurial career. In this regard, the following hypothesis was shaped.

*H2: Role models influence one*’s *entrepreneurial self-efficacy.*

### 3.3 Entrepreneurial perceived behaviour control

Both researchers and educators have demonstrated that perceived self-efficacy influences behaviour. Entrepreneurial self-efficacy plays a critical role in motivating individuals to become entrepreneurs. Fundamentally, Bandura (1986) concluded in his empirical study on entrepreneurial education and self-efficacy that entrepreneurial education positively affects one’s perceptions of his or her ability to become an entrepreneur. However, until now only a few studies have examined the antecedents of entrepreneurial intentions and nascent PBC as career choices in entrepreneurship (education) research (e.g., Zhao et al., 2005; Barbosa et al., 2007; McGee et al., 2009; Zellweger et al., 2011). Entrepreneurial intentions have been used as self-predictions of expected behaviour (Ajzen, 1991; Ajzen and Fishbein, 1977). In short, once intentions are developed, real behaviour can be expected.

Several empirical studies have focused on nascent entrepreneurship (e.g., Carter et al., 2003; Davidsson and Honig, 2003; Arenius and Minniti, 2005). However, only a few have included entrepreneurial self-efficacy as an explanatory variable of nascent behaviour (McGee et al., 2009). Nevertheless, the theory linking entrepreneurial self-efficacy and entrepreneurial nascent behaviour is rather straightforward. Because nascent behaviour follows intentions, influences that encourage intention – including entrepreneurial self-efficacy – would likewise explain nascent entrepreneurial behaviour. In this regard, as research shows that self-efficacy plays a significant role in determining career choices (Chen et al., 2004; Pihie and Bagheri, 2013; Saeed et al., 2015), self-efficacy reflects an appropriate construct for explaining behaviour. Furthermore, PBC can be modified by providing entrepreneurial perspectives and knowledge regarding entrepreneurial experiences (Van Gelderen et al., 2008). In short, entrepreneurship education with storytelling shows great potential to boost PBC by enhancing one’s perspective about his or her capability to engage in entrepreneurial activities. In line with this argumentation, the following hypothesis was proposed.

H3: Entrepreneurial self-efficacy increases PBC.

The model in the present study undertakes a causal chain from entrepreneurial stories to role models to the intervening concepts, including self-efficacy, and finally to the central result of entrepreneurship education: entrepreneurial PBC (Figure 1). In other words, an individual’s perceptions and PBC will be altered positively by observing role models via multimedia entrepreneurship education, and this modification in perceptions will significantly increase entrepreneurial PBC.

## 4 Methodology

### 4.1 Research design

Data for the present study were collected in Austria, Finland, and Greece from February–July, 2016. The participants volunteered to participate in an entrepreneurship education course outside the curriculum and were free to choose which story from which entrepreneur they would watch. The measurement instrument was a questionnaire, which participants completed both before and after viewing the entrepreneurial story from the role model. The study included only pairs of completed questionnaires. Although the study used an ex-ante-ex-post-research design, it used only the results of the post-assessment, after participants had watched an entrepreneurial role model tell a story online, which was an entrepreneurship education activity outside the curriculum and was designed especially for this study.

### 4.2 Sample

The study’s research sample included 160 individuals in Austria, 128 in Finland, 103 in Greece, and 35 participants in other locations, for a total of 426 participants, 37.32% of whom were female and 62.68% of whom were male. The course consisted of seven entrepreneurial stories told by entrepreneurs from Austria, Finland, and Spain who had founded small and medium-sized companies in Austria, Finland, Spain, Italy, Germany, Switzerland, the United States of America, and Australia. The business sectors involved included venture capital (the story watched by 73 individuals), information technology services (watched by 91 individuals), transportation services (watched by 85 individuals), energy production (watched by 103 individuals), tea production and trading (watched by 48 individuals), export advice (watched by 21 individuals), and tax advice (watched by 5 individuals). The participants were free to choose which video to watch. The distribution was robust, which was essential to the analysis. Participants were in the following age ranges: under 18 years (31 participants), from 18–24 years (313 participants), from 25–34 years (51 participants), from 35–44 years (18 participants), from 45–55 years (11 participants), and over 56 years (2 participants). While 113 participants had already participated in some form of entrepreneurship education, 313 reported no prior participation in entrepreneurship education of any kind.

### 4.3 Measurements

In general, the success or failure of variables was measured using statements made by the entrepreneurs during their videos. To measure the stories’ effectiveness, two categories of the statement were created, positive and negative, and statements from each video were categorised as one or the other. Participants were asked to classify the statements for true or wrong.

#### Success and failure stories

The entrepreneurs highlighted the successes and failures in their entrepreneurial lives with particular statements. If participants remembered the statements correctly after watching the story, the statement was assigned a value of 1. Prior research was taken into account when applying this method of scoring narratives (e.g., Harmeling and Sarasvathy, 2013). Table 4 in the Appendix presents the statements and their associated scores, including the means and standard deviations (SD).

#### Role models

Participants were asked if they had an entrepreneurial role model and, if so, who it was. They were asked to evaluate their entrepreneurial role models, including four items entitled ‘parents or siblings’, ‘friends’, ‘someone else who is important to me and/or someone I do not know personally’, and ‘the entrepreneur from the video’ on a seven-point scale, with 1 meaning completely disapprove of and 7 meaning completely approve. The Cronbach’s alpha for this measure was 0.74. Table 5 in the Appendix shows the mean, SD, item total correlation, and the Cronbach’s alpha if the item was deleted.

#### Self-efficacy

Participants indicated their level of agreement (from 1 for strongly disagree to 7 for strongly agree) with 10 statements related to various tasks required to become an entrepreneur. For instance, based on items identified by Chen et al. (2001) and Kickul et al. (2009) regarding the search stage of entrepreneurship, a participant stated their agreement regarding Task 1, which was to conceive a unique idea for a business. Other tasks measured the marshalling stage and the implementation stage. In principle, all the items reflected the entrepreneurial self-efficacy variable, which relates to “one’s estimate of one’s overall ability to perform successfully in a wide variety of achievement situations, or to how confident one is that she or he can perform effectively across different tasks and situations” (Chen et al., 2001, 63). The Cronbach’s alpha for this measure was 0.938. Table 6 in the Appendix lists the tasks, including the means, SD, item total correlation, and the Cronbach’s alpha if the task was deleted.

#### Perceived behavioural control (PBC)

Based on items identified by Liñán and Chen (2009), participants indicated their level of agreement (with 1 indicating strong disagreement and 7 indicating strong agreement) with statements regarding their entrepreneurial capacity. The present study assessed PBC by the extent to which a person rated founding a business as feasible and the extent to which he or she felt well prepared. To this end, the study rated 6 items measuring PBC on a 7-point Likert scale. The Cronbach’s alpha for this measure was 0.935. Table 7 in the Appendix contains the items, including the means, SD, item total correlation, and the Cronbach’s alpha if the item was deleted.

### 4.4 Control variables

Prior studies have found significant gender-related differences in perceived entrepreneurial self-efficacy and PBC (e.g., Martin et al., 2013; Shinnar et al., 2014) in the context of entrepreneurship education and other frameworks related to entrepreneurial orientation (e.g., Powell and Eddleston, 2013; Goktan and Gupta, 2013; Kelley et al., 2016; Fellnhofer et al., 2016). Thus, the present study controlled for gender. In addition, age has been found to be related to both PBC (e.g., Rauch and Hulsink, 2015) and entrepreneurial self-efficacy (e.g., Vázquez et al., 2011), which led to the decision to control for age, as it was expected that the course format used in the study did not suit all age groups equally well. Furthermore, entrepreneurial experience was controlled for and evaluated with yes or no questions based on those suggested by Peterman and Kennedy (2003), which asked whether the participant, parents, other family members, friends, or another person important to the participant had ever started a business and whether the participant had any work experience in a small or new business or in self-employment or as an entrepreneur. Finally, it has been controlled for nationality during the course of this research in the examination of 426 individuals from Austria, Finland and Greece to identify potential differences in different countries.

### 4.5 Data validity and reliability

Table 1 illustrates the results of a confirmatory factor analysis (CFA) summarising eight checks, labelled (a)–(h), for validity and reliability. As shown in (a), all standardised factor loadings (SFLs) were significant (*t* > 3.1; *p* < 0.001). As shown in (b), based on Bagozzi and Baumgartner (1994), the indicator reliability (IR) of all items was > 0.4. As shown in (c), according to Bagozzi and Yi (1988) and Raykov (1997), the composite reliability (CR) of the variables was > 0.6. As shown in (d), all Cronbach’s alphas were >0.7, as recommended by Nunnally (1978) and Hair et al. (1995). As shown in (e), according to Fornell and Larcker (1981), the average amount of variance (AVE) reflected was >0.5. As shown in (f), all Kaiser-Meyer-Olkin Measures of Sampling Adequacy (KMO) were >0.5 (Kaiser, 1974). As shown in (g), all determinants of the constructs’ correlation matrices were >0.00001. The variable self-efficacy is below but with respect to the robustness of all the other indicators, this value can be neglected. As shown in (h), all significant values expressing the results of Bartlett’s Test of Sphericity (BTS) showed suitable correlations in the dataset (Bartlett, 1937).

## 5 Results

Table 2 shows the construct means, SD, and correlations of all study variables and shows that the means were equal for stories of success and failure. In accordance with the recommendations of Tabachnick and Fidell (1996), the bivariate correlation between the independent variables did not exceed 0.70. Thus, multicollinearity does not appear to have been a critical issue in this research. In addition, the variance inflation factor (VIF) was below the acceptable level of 2.5 (Baguley, 2012).

In addition, the data (*n* = 426) were analysed using linear regression examinations for the model. Table 3 shows the results. First, Hypothesis 1 (H1) proposed that observing either entrepreneurial success stories (H1a) or entrepreneurial failure stories (H1b) in the course of a web-based entrepreneurship training program would influence perceptions of role models. The results indicated that neither stories of success (*B* = –0.886, n.s.) nor stories of failure (*B* = 0.573, n.s.) significantly influenced the participants’ perceptions of roles models. Therefore, H1 was not supported. In addition, it did not matter whether role models shared negative or positive experiences with nascent entrepreneurs. However, the results did support hypothesis 2 (H2), demonstrating that role models do have a significant positive influence on entrepreneurial self-efficacy (*B* = 0.230***, *p* < 0.01). Hypothesis 3, which proposed that exposure to entrepreneurial self-efficacy would increase entrepreneurial PBC, was also supported because a significant positive effect was found (*B* = 0.702***, *p* < 0.01). While nationality appears to have no significant influence of the variables under investigation, entrepreneurial experience and gender appear to play a significant role. The higher the experience, the higher is the impact of role models, entrepreneurial self-efficacy and PBC. Furthermore, males tend to perceive entrepreneurial self-efficacy and PBC higher than females after observing entrepreneurial stories.

**Figure 2:**
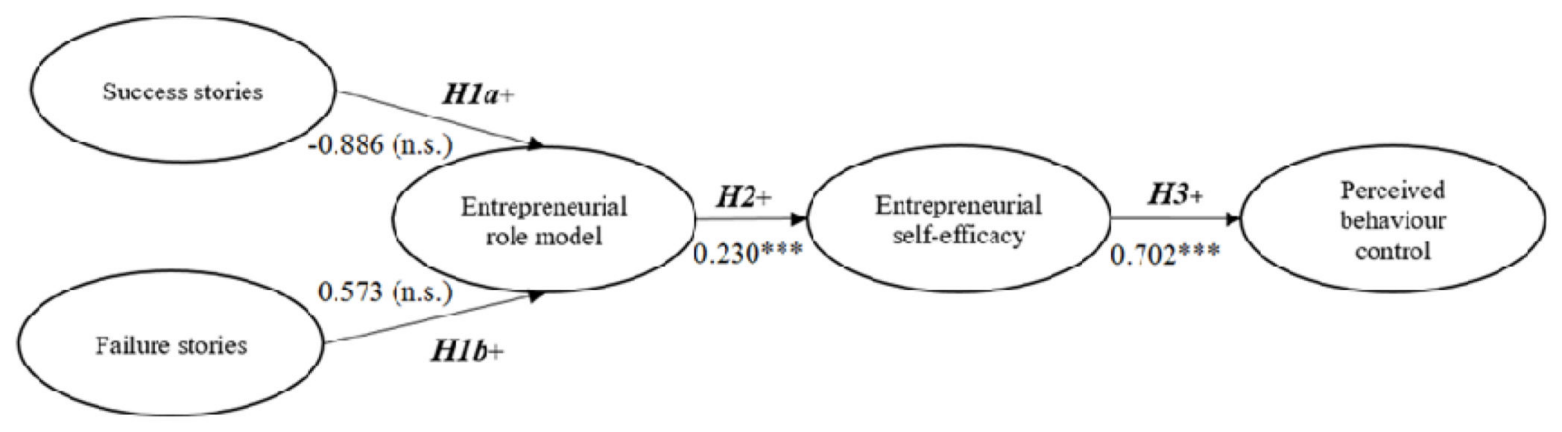
Research model results with unstandardised coefficients *B* Significance code: ***p < 0.01.

Finally, as the results of the linear regression analysis indicated, the research model, which assumed a causal chain from role models’ entrepreneurial stories to the intervening concepts, including self-efficacy, had a significant positive effect on the results of entrepreneurship education about entrepreneurial PBC (Figure 2). Overall, there is no significant effect on the perception of role models when potential entrepreneurs listen to success (*B* = –0.886, n.s.) or failure stories (*B* = 0.573, n.s.) of entrepreneurs. In other words, it does not matter whether the role models observed focus on the positive or negative aspects of their entrepreneurial experiences. However, overall participants’ perceptions of entrepreneurial self-efficacy were significantly positively affected by observing role models (*B* = –0.230***) in the context of multimedia entrepreneurship education, and this effect on perception significantly increased entrepreneurial PBC (*B* = 0.702***).

Baron and Kenny (1986) proposed a four step approach in which several regression analyses are conducted for testing mediation with regression analysis. Because there are significant relationships from entrepreneurial role models through perceived behaviour control, it has been proceeded to test mediation with regression analysis. This holds true in this model. In the fourth step, mediation is supported if the effect of entrepreneurial self-efficacy remains significant after controlling for entrepreneurial role models. Because entrepreneurial role model is still significant (*B* = 0.187***) when entrepreneurial self-efficacy is controlled (*B* = 0.632***), the finding supports partial mediation (adjusted *R* Square = 0.510; *F* = 50.193***).

## 6 Discussion and conclusions

The central purpose of this study was to investigate the effects of entrepreneurial role models on entrepreneurship education using the TPB (Ajzen, 1988), social learning theory (Bandura, 1986) and Dyer’s (1994) model of entrepreneurial careers. The study aimed to fill a gap in research on the effects of roles models on entrepreneurial PBC. Results showed that role models in entrepreneurship education had a significant positive effect on participants’ self-efficacy and PBC to become an entrepreneur.

Results of the present study will contribute threefold to the ongoing debate in the entrepreneurship-education literature. First, the findings emphasised that Ajzen’s fundamental theory (1988) offers a valuable framework for understanding the effects of role models in entrepreneurship education about entrepreneurial PBC. The present study enhanced the existing academic discussion by showing that observing role models affect perceptions. Within this framework, this study highlighted the content in multimedia storytelling and its effects in the course of entrepreneurship education. It made no difference whether entrepreneurs stressed the negative or positive aspects of the entrepreneurial life or both. This understanding will aid the design of effective, practical entrepreneurship courses (Edelman et al., 2008).

Second, the present study demonstrated that role models in entrepreneurship education affected entrepreneurial PBC via self-efficacy, the effect of which was significantly positive. Therefore, entrepreneurial role models deserve more of a role in entrepreneurship education designed to give students a realistic picture of being an entrepreneur. This engagement need not necessarily focus on entrepreneurship’s positive or negative aspects, but it should motivate and inspire participants. For instance, several researchers have noted that emotions and passion strongly enhanced entrepreneurship education (e.g., Souitaris et al., 2007; Bhoyar and Nagendra, 2014; Lyons et al., 2015), as they are essential to the entrepreneurial temperament (Cardon et al., 2013).

Finally, while the present study provided no recommendations regarding the most suitable course design to ensure entrepreneurship education that stimulates entrepreneurial PBC, it provided evidence that entrepreneurial role models increased entrepreneurial PBC via self-efficiency. Furthermore, integrating role models in the curriculum or merge them with other entrepreneurship-education curriculum design, for instance, with game-based learning (e.g., Xinaris et al., 2011; Fellnhofer, 2015), opportunity identification (DeTienne and Chandler, 2004), entrepreneurial action (Neck and Greene, 2011), or out of a diverse teaching pool (Lorz et al., 2013; Gedeon, 2014) shows great potential.

### 6.1 Limitations

The results of the present study must be interpreted with caution. First, the study used only the results of a post-assessment questionnaire on a web-based entrepreneurial program. In addition, the generalisability of its findings may be limited by regional and national differences in PBC, as highlighted by prior studies (e.g., Bernhofer and Han, 2014). In addition, this study illuminates light on the influence of different role models outside the classroom. Thus, to which extent these effects could be integrated into the curriculum requires further research effort. Furthermore, its results regarding the type of web-based entrepreneurship education used must be compared to the results of other research designs. Furthermore, when explaining and demonstrating the mediating role of entrepreneurial self-efficacy in the effect of entrepreneurial role model on perceived behavioural control, bootstrapping mediation analysis could be also used. A bootstrapping approach to mediation analyses would allow further findings when testing the significance of the indirect effects (Hayes, 2009; Shrout and Bolger, 2002). Finally, the study did not include all elements of the TPB, which leaves room for further research, as do the complexities of entrepreneurship education in general.

### 6.2 Future research

Various future research avenues present themselves. Although the present study confirmed that role models in the context of entrepreneurship education increased entrepreneurial PBC, future research is required to inspect the contextual issues in more detail than the present framework allowed. For instance, analysing the mediating and moderating effects using structural equation models would provide further interesting insights into how entrepreneurial PBC is affected at various levels.

### 6.3 Practical implications

From a practical viewpoint, entrepreneurship education aims to have a positive effect on potential entrepreneurs, increasing their motivation to choose such a non-traditional career path regardless of age, gender, education, and social or cultural background. Entrepreneurship education is as complex and multifaceted as entrepreneurship itself. At its heart, entrepreneurship education should illuminate a promising, valuable path for potential entrepreneurs. Consequently, entrepreneurship education must go beyond the transfer of knowledge and understanding of theoretical constructs to shape future reality in practice (Neck and Greene, 2011). This study proposed one element for entrepreneurship education and raised valuable issues that must be considered in future research. Meanwhile, both educational and social institutions must identify ways to become more supportive of entrepreneurial creativity, inspiration, and innovation (Walter and Block, 2016).

## Supplementary Material

Appendix

## Figures and Tables

**Table 1:** Confirmatory factor analysis (CFA) results

*Construct*	*Items*	(*a*) *SFL*	(*b*) *IR ≥ 0.4*	(*c*) *CR ≥ 0.6*	(*d*) *Cronbach’s α ≥ 0.7*	(*e*) *AVE ≥ 0.5*	(*f*) *KMO*	(*g*) *D ≥ 0.00001*	(*h*) *BTS*
Role model	RM_1	0.664	0.441	0.755	0.74	0.652	0.684	0.365	426.056
	RM_2	0.886	0.785						
	RM_3	0.548	0.3						
	RM_4	0.511	0.261						
Self-efficacy	SE_S_1	0.724	0.524	0.938	0.938	0.777	0.91	0.000	3362.571
	SE_S_2	0.818	0.669						
	SE_P_1	0.844	0.713						
	SE_P_2	0.765	0.585						
	SE_M_1	0.755	0.57						
	SE_M_2	0.771	0.594						
	SE_M_3	0.702	0.492						
	SE_M_4	0.805	0.648						
	SE_I_1	0.782	0.611						
	SE_I_2	0.8	0.64						
Perceived behavioural control	BC_1	0.815	0.664	0.932	0.935	0.833	0.895	0.006	2129.336
	BC_2	0.861	0.741						
	BC_3	0.892	0.796						
	BC_4	0.807	0.651						
	BC_5	0.826	0.682						
	BC_6	0.797	0.636						

**Table 2:** Construct means, SD, and correlations of study variables

		*Mean*	*SD*	*1*	*2*	*3*	*4*	*5*
1	Role model	4.00	1.31	1				
2	Self-efficacy	4.43	1.17	0.295**	1			
3	Success stories	0.08	0.06	–0.014	0.086	1		
4	Failure stories	0.08	0.06	0.049	0.102*	0.603**	1	
5	Perceived behaviour control	3.67	1.38	0.252**	0.656**	0.041	0.044	1

*n* = 426; Pearson correlation (bivariate); standard deviation (SD).

* Correlation is significant at the 0.05 level (2-tailed).

** Correlation is significant at the 0.01 level (2-tailed).

**Table 3:** The model’s linear regression results (*n* = 426)

	*H1*				*H2*		*H3*	
	
	*Role model*				*Entrepreneurial self-efficacy*		*Perceived behaviour control*	
	
	*B*	*SE*	*B*	*SE*	*B*	*SE*	*B*	*SE*
*Independent variables*								
(constant)	3.707***	0.334	3.637***	0.336	2.734***	0.318	–0.879***	0.298
Success stories	–0.886	1.069						
Failure stories			0.573	1.042				
Role model					.230***	0.041		
Entrepreneurial self-efficacy							0.702***	0.043
*Control variables*								
Entrepreneurial experience	1.095***	0.260	1.058***	0.260	1.054***	0.223	0.807***	0.207
Age	–0.157*	0.082	–0.155*	0.082	–0.021	0.070	0.222***	0.063
Gender	–0.011	0.131	–0.023	0.131	.317***	0.111	0.367***	0.102
Nationality	0.027	0.021	0.027	0.021	0.010	0.018	–0.014	0.016
Field of study	0.016	0.027	0.012	0.027	–0.027	0.023	–0.013	0.021
Entrepreneur watched	0.024	0.047	0.025	0.047	–0.032	0.039	0.023	0.036
*R-Square*	0.053		0.052		0.158		0.494	
*Adjusted R-Square*	0.037		0.036		0.144		0.486	
*F*	3.330**		3.272***		11.217***		58.311***	

Standard error (SE), unstandardised coefficients B (B), Significance codes: *p < 0.1, **p < 0.05, ***p < 0.01.
