# Supplementary material for: Entrepreneurship education revisited: perceived entrepreneurial role models increase perceived behavioural control
Source: Int J Learn Chang. Author manuscript; Available in PMC 2017 Nov 2. (PMC5667741; doi:10.1504/IJLC.2017.086856)
Supplement: Appendix [file NIHMS74614-supplement-Appendix.pdf]

Appendix

Table 4 Items for success and failure stories

We would like to know what do remember of the videos. Which citations are from which entrepreneurs?

Citations from success stories	*Mean*	*SD*	*Citations from failure stories*	*Mean*	*SD*
Important skills are goal orientation, faith and perseverance	0.09	0.285	Quitting is failure	0.06	0.235
Never get started without a mission!	0.08	0.275	Always keep an eye on your cash flow!	0.07	0.252
Define your own success indicators!	0.07	0.248	There is no right or wrong decision.	0.10	0.302
Just do it – Live your dream! Success is to do and make your dreams come true	0.09	0.292	You should always have an eye on the market and have a diverse range of products or service to find your cash cows of tomorrow!	0.12	0.328
Get the real good customers and get recognised by competitors. It is the passion and the strong will to do something besides talking to the customers to feel their need	0.08	0.278	Do not underestimate the power of marketing!	0.15	0.358
It is all about the customer. Try to grab the customer and learn with him to develop your business	0.12	0.322	Failures are part of the business - it is a trial and error issue daily	0.10	0.302
Independence of my whole doing was always important	0.01	0.108	Maybe I have started my company too early and too young	0.01	0.084
The good motivator is behind the money	0.01	0.108	The first years were really hard for me	0.01	0.118
The most important is to be awake and to think a lot	0.01	0.097	To be an entrepreneur needs a lot of time and a lot of energy	0.02	0.144
Independency and realising own ideas are great reasons for starting your business	0.03	0.178	Keep your eyes open for new entrepreneurial opportunities even it is not easy to evaluate them.	0.04	0.207
Honesty is important for a long term success	0.04	0.201	It is important to evaluate the resources for exploring and exploiting entrepreneurial opportunities	0.04	0.185
At the beginning it is important to have a mission!	0.04	0.201	It is important to have enough time for your preparation before starting the business	0.04	0.207
Einzigartiges Service und den täglichen Überblick haben. Kunden sollen kommen um zu bleiben	0.10	0.302	Entscheidungen müssen getroffen werden.... Immer auf die eigenen Ressourcen achten	0.09	0.292

Table 4 Items for success and failure stories (continued)

We would like to know what do remember of the videos. Which citations are from which entrepreneurs?

Citations from success stories	*Mean*	*SD*	*Citations from failure stories*	*Mean*	*SD*
Gesundes Selbstbewusstsein ist essentiell	0.11	0.311	Als Unternehmer bist du für alles verantwortlich! Wenn es hart auf hart geht, ist man als Unternehmer der Letzte der überbleibt	0.09	0.289
Immer mit offenen Augen und Ohren durch das Unternehmen und das Leben gehen um neue unternehmerischen Möglichkeiten zu erkennen	0.10	0.302	Geht nicht, gibt es nicht. Es gibt immer einen Weg!	0.11	0.317
Mein Leben ist begeisterungsfähig, verantwortungsvoll und nachhaltig geworden. Mein privates Leben wurde zu 100% positiv beeinflusst	0.08	0.264	Als arbeitsloser Bauer wollte ich kein Wirtschaftsflüchtling werden und unterstützte die Bauern, die keinen Absatz hatten, da ich keine Arbeit hatte	0.08	0.264
Selbst entscheiden was ich wann tue. Wenn man etwas für sich selbst macht, geht es schneller und besser zum Erfolg	0.08	0.264	Der Hauptgrund mich selbstständig zu machen war die fehlende Wertschätzung als Unselbstständiger	0.07	0.248
Ich möchte authentisch bleiben und durch das entgegengebrachte Vertrauen Arbeitsplätze schaffen	0.06	0.244	Wichtig ist die Leute zu fragen, was sie eigentlich brauchen! Unsere Kunden liefern uns Ideen und unsere zukünftigen Innovationen	0.06	0.240
It is always important to improve the technology in order to create local value and local jobs and to find new opportunities	0.08	0.271	A failure occurs when an unseen situation occurs	0.08	0.268
The project is exciting. Meeting new people is great. You can enrich your life a lot with entrepreneurial activities	0.17	0.375	When you have failed, you have to learn from them. You can learn a lot. Try to learn!	0.15	0.355
Success is to meet targets at 100%	0.13	0.336	Analyse different business scenarios, include all different stakeholders and work with a good team to prevent mistakes	0.12	0.322

Table 5 Role model items

If you have an entrepreneurial role model, who is it?

Items (scale: 1= total disapproval....... 7= total approval)		*Mean*	*SD*	*Item total correlation*	*Alpha if item is deleted*
RM_1	Parents or siblings	3.94	1.928	0.508	0.699
RM_2	Friends	3.96	1.706	0.693	0.587
RM_3	Someone else who is important to me and/or someone I do not know personally	4.34	1.691	0.472	0.713
RM_4	The entrepreneur from the video	3.76	1.638	0.474	0.712

Table 6 Self-efficacy items based on Chen et al. (2001) and Kickul et al. (2009)

Please indicate your level of agreement with the following statements!

Items (scale: 1 = not confident; 7 = completely confident)		*Mean*	*SD*	*Item total correlation*	*Alpha if item is deleted*
Searching stage					
SE_S_1	Task 1: Conceive a unique idea for a business	1.595	1.595	0.934	0.934
SE_S_2	Task 2: Identify market opportunities for a new business	1.492	1.492	0.929	0.929
Planning stage					
SE_P_1	Task 3: Plan a new business	1.545	1.545	0.928	0.928
SE_P_2	Task 4: Write a formal business plan	1.577	1.577	0.932	0.932
Marshalling stage					
SE_M_1	Task 5: Raise money to start a business	1.593	1.593	0.932	0.932
SE_M_2	Task 6: Convince others to invest in your business	1.543	1.543	0.930	0.930
SE_M_3	Task 7: Convince a bank to lend you money to start a business	1.609	1.609	0.934	0.934
SE_M_4	Task 8: Convince others to work for you in your new business	1.455	1.455	0.929	0.929
Implementing stage					
SE_I_1	Task 9: Manage a small business	1.511	1.511	0.932	0.932
SE_I_2	Task 10: Grow a successful business	1.472	1.472	0.931	0.931

Table 7 Perceived behavioural control based on Liñán and Chen (2009)

To what extent do you agree with the following statements regarding your entrepreneurial capacity?

Items (scale: 1= strongly disagree....... 7= strongly agree)		*Mean*	*SD*	*Item total correlation*	*Alpha if item is deleted*
BC_1	To start a firm and keep it working would be easy for me	3.56	1.589	0.765	0.928
BC_2	I am prepared to start a viable firm	3.54	1.645	0.830	0.920
BC_3	I can control the creation process of a new firm	3.85	1.547	0.841	0.919
BC_4	I know the necessary practical details to start a firm	3.51	1.606	0.822	0.921
BC_5	I know how to develop an entrepreneurial project	3.51	1.627	0.837	0.919
BC_6	If I tried to start a firm, I would have a high probability of succeeding	4.01	1.498	0.752	0.930
